# Regulation of Glutathione Peroxidase 4 in Diabetic Retinopathy

**DOI:** 10.3390/ijms27177815

**Published:** 2026-08-31

**Authors:** Pooja Malaviya, Renu A. Kowluru

**Affiliations:** Department of Ophthalmology, Visual and Anatomical Sciences, Wayne State University, Detroit, MI 48201, USA

**Keywords:** diabetic retinopathy, ferroptosis, glutathione peroxidase, mitochondria, posttranslational modification, retina, ubiquitination

## Abstract

Oxidative stress-mitochondrial dysfunction plays a central role in diabetic retinopathy. Glutathione peroxidase 4 (GPx4), a key antioxidant enzyme that detoxifies lipid peroxides, is inhibited in diabetes, but the mechanism underlying its inhibition is unclear. Here, we have investigated the role of ubiquitination in retinal GPx4 inhibition in diabetes. Primary human retinal endothelial cells and Müller cells, incubated in 20 mM (high) D-glucose, were analyzed for GPx4 activity and its ubiquitination. GPx4 interaction with ubiquitin ligase TRIM46 and with mitochondrial membrane transporters was analyzed by immunofluorescence and by co-IP. The role of TRIM46 in mediating GPx4 ubiquitination and mitochondrial membrane damage was confirmed using *TRIM46*-siRNA. Results were confirmed in the retina from streptozotocin-induced diabetic mice and from human donors with documented diabetic retinopathy. Compared to cells in normal glucose, high glucose significantly increased GPx4 ubiquitination in both retinal endothelial and Müller cells, inhibited cytosolic and mitochondrial GPx4 activity by >30%, and upregulated TRIM46 expression and TRIM46-GPx4 interactions. High glucose had no effect on TRIM46 mitochondrial import, but GPx4 import was decreased, and GPx4 in the mitochondria was more ubiquitinated; TRIM46-siRNA prevented the decrease in cytosolic and mtGPx4 activities. Similarly, retina from diabetic mice and human donors with diabetic retinopathy had increased TRIM46 and ubiquitinated GPx4 and decreased GPx4 activity. Diabetes induces TRIM46-mediated ubiquitination of GPx4 in the cytosol and impairs its mitochondrial import, leading to mitochondrial damage and cell death.

## 1. Introduction

Diabetes affects the whole body, and in the eye, retinopathy is the major cause of vision loss in working-age adults, affecting over 80% of patients with diabetes for 20 years [1,2,3]. However, in the non proliferative stages of the disease, most of the patients are asymptomatic, but as the disease progresses, new abnormal blood vessels begin to grow, and, if neovascularization is not controlled, proliferative diabetic retinopathy could lead to blindness [1]. High glucose triggers several metabolic, molecular, and functional abnormalities in the retina, including an increase in oxidative stress and inflammation, and mitochondria become dysfunctional, culminating in vascular and nonvascular cell damage/loss [1,4,5,6]. Although cells have a very efficient antioxidant defense system, including catalase, superoxide dismutase, glutathione peroxidase, and glutathione (GSH), sustained hyperglycemia-mediated metabolic and molecular abnormalities overwhelm the system, resulting in increased oxidative stress and mitochondrial damage and leading to retinal cell death [5,7,8].

Retina is a lipid-rich, highly metabolic tissue with 1/3rd of its dry weight represented by lipids [9], and its membranes are enriched in polyunsaturated fatty acids (PUFAs) that help maintain membrane biophysical properties. However, PUFAs can readily be attacked by free radicals, forming lipid peroxides [10]. Mitochondria are also very rich in lipids that help maintain mitochondrial membrane integrity, and PUFAs in mitochondrial membranes are also the primary targets for ROS attack, leading to lipid peroxidation [11]. Lipid peroxides are detoxified by the selenoprotein glutathione peroxidase 4 (GPx4), which uses GSH as a cofactor, and GPx4 is also ubiquitously present in the inner membrane of the mitochondria [12,13]. In diabetic retinopathy, GPx4 activity is decreased in the cytosol and in the mitochondria, leading to endothelial cell loss by ferroptosis, an iron-dependent programmed cell death, which is intimately associated with lipid peroxide accumulation [14,15]. However, the mechanism of inhibition of GPx4 in diabetes is unclear.

Enzyme function can be modulated by posttranslational modifications, and ubiquitination is one of the major covalent modifications that regulates protein stability and subcellular distribution. It ultimately renders proteins for degradation by the proteasome, affecting intracellular protein levels [16,17]. Ubiquitination of GPx4 is shown to affect its functional activation, inducing cell loss; e.g., in non-small cell lung cancer cells, a GPx4 inhibitor accelerates cell loss by ubiquitinating it [18,19]. In diabetic retinopathy, ubiquitination of mitochondrial transcription factor TFAM is associated with suboptimal mtDNA biogenesis and instability [20]; however, whether ubiquitination contributes to inhibition of GPx4 in diabetes is unclear.

Based on these, we proposed the hypothesis that hyperglycemia promotes GPx4 ubiquitination, impairing its mitochondrial translocation and inhibiting its activity. The hypothesis was tested using in vitro (retinal endothelial cells and Müller cells) and in vivo (retina from diabetic mouse) models, and the key results were validated in the retina from human donors with documented retinopathy. Retinal endothelial and Müller cells, representing vascular and glial cells, respectively, were selected because while histopathology characteristic of diabetic retinopathy is observed in the retinal vasculature, Müller cells are the most common glial cells that expand across almost the whole width of the retina and envelop retinal capillaries, forming anatomical connections between the neurons and blood vessels [2,21,22], and both these cells have dysfunctional mitochondria and accelerated cell death in diabetes. The results presented here clearly show that E3 ubiquitinase TRIM46, which facilitates the transfer of ubiquitin from an E2 enzyme to the substrate, plays a major role in GPx4 ubiquitination, and despite increased levels of ubiquitinated GPx4 in the mitochondria, TRIM46 levels are not increased, suggesting that GPx4 could be ubiquitinated in the cytosol, and due to poor interactions of the ubiquitinated GPx4 with mitochondrial transporters, its import inside the mitochondria is decreased.

## 2. Results

### 2.1. In Vitro

**Figure 1:**
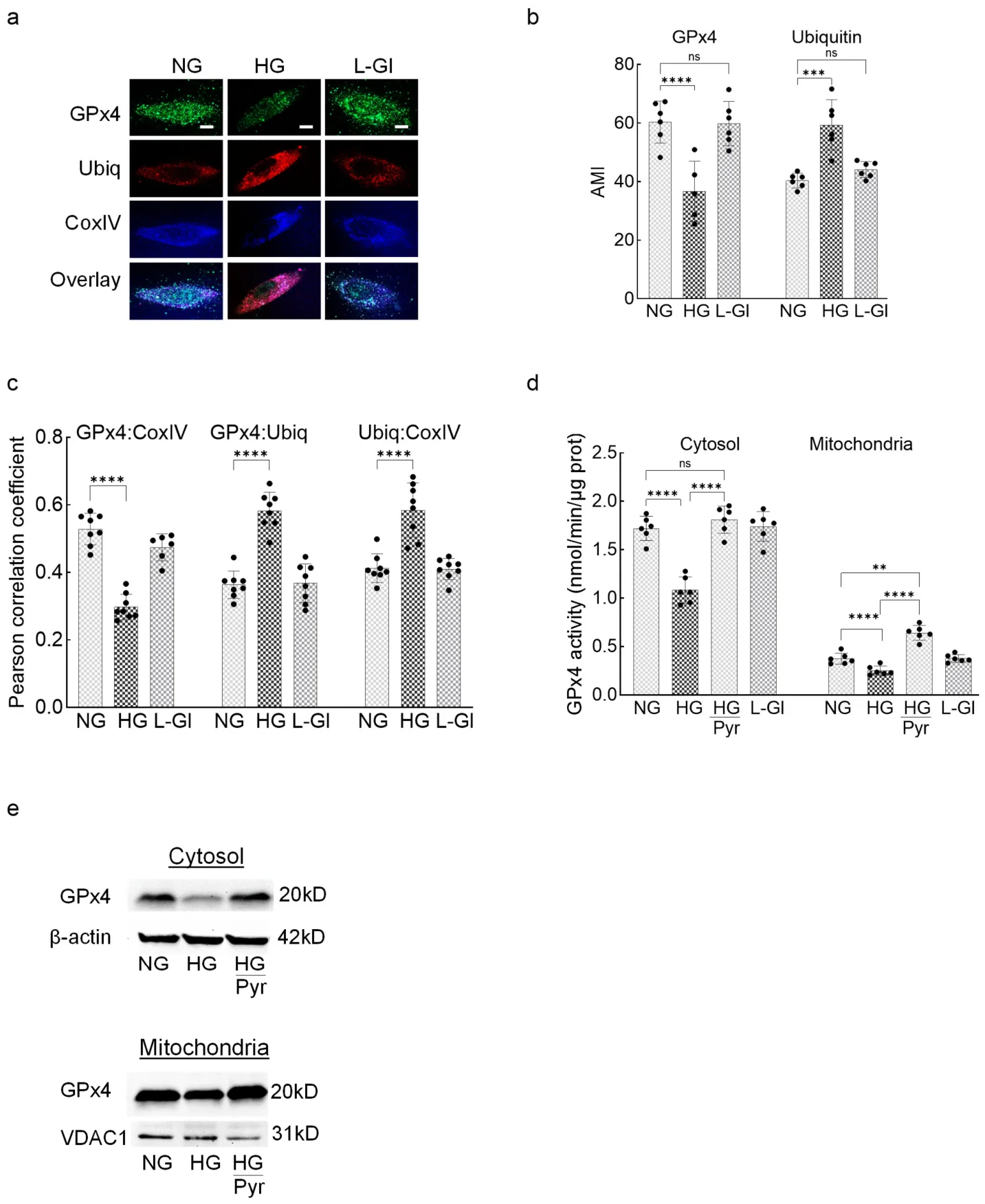
Effect of high glucose on GPx4 ubiquitination. HRECs incubated in high glucose for 96 h were analyzed for (**a**) GPx4 mitochondrial localization and ubiquitination. Representative immunofluorescence image of HRECs, captured with a 63× objective, displays GPx4 (green), ubiquitin (red), and CoxIV (blue); scale bar = 10 µm. Plots showing (**b**) AMI of GPx4 and ubiquitin and (**c**) Pearson correlation coefficients between GPx4-CoxIV, GPx4-ubiquitin, and ubiquitin-CoxIV. (**d**) GPx4 activity was quantified in cytosolic and mitochondrial fractions. (**e**) GPx4 protein expression in cytosol and mitochondria was determined by Western blotting; β-actin and VDAC1 served as their respective loading controls. Data in the graphs are represented as mean ± SD from three or more experiments, and for immunocytochemistry, 5–10 randomly selected fields per slide were quantified. NG and HG = 5 mM or 20 mM D-glucose, respectively; L-Gl = 20 mM L-glucose; HG/Pyr = HRECs pre-incubated with PYR 41, followed by incubation in 20 mM D-glucose. ** *p* < 0.01; *** *p* < 0.001; **** *p* < 0.0001; ns = *p* > 0.05.

Human retinal endothelial cells (HRECs): As hyperglycemia inhibits retinal GPx4, a phospholipid hydroperoxidase [14], to understand the mechanism of GPx4 inhibition, the role of ubiquitination was determined. Compared to normal glucose (5 mM D-glucose), total GPx4 expression and its arithmetic mean intensity (AMI) were decreased in cells in high glucose (20 mM D-glucose), and mitochondrial expression of GPx4 was also reduced, as evidenced by a strong Pearson correlation coefficient between GPx4 and CoxIV (>0.5) in normal glucose, vs. weak (~0.3) in high glucose. In the same cells, ubiquitin expression was also significantly increased. Although the correlation between GPx4-ubiquitin in cells in normal glucose was weak (~0.3), it was strong (>0.5) in cells in high glucose, suggesting increased ubiquitination. However, incubation of cells in 20 mM L-glucose (osmotic control), instead of 20 mM D-glucose, had no effect on GPx4 expression and its mitochondrial localization. Consistent with cytosolic GPx4, the Pearson correlation between CoxIV and ubiquitin was not strong in cells in normal glucose but was significantly higher in cells in high glucose, suggesting increased ubiquitin inside the mitochondria (Figure 1a–c). To examine if GPx4 inhibition is due to increased ubiquitination, enzyme activity was measured in the cytosol and mitochondria. Ubiquitination inhibitor PYR 41 (4-(4-(5-Nitro-furan-2-ylmethylene)-3,5-dioxo-pyrazolidin-1-yl)-benzoic acid ethyl ester) ameliorated the glucose-induced decrease in GPx4 expression in both cytosol and mitochondria and ameliorated inhibition of cytosolic and mitochondrial GPx4 activities (Figure 1d,e).

**Figure 2:**
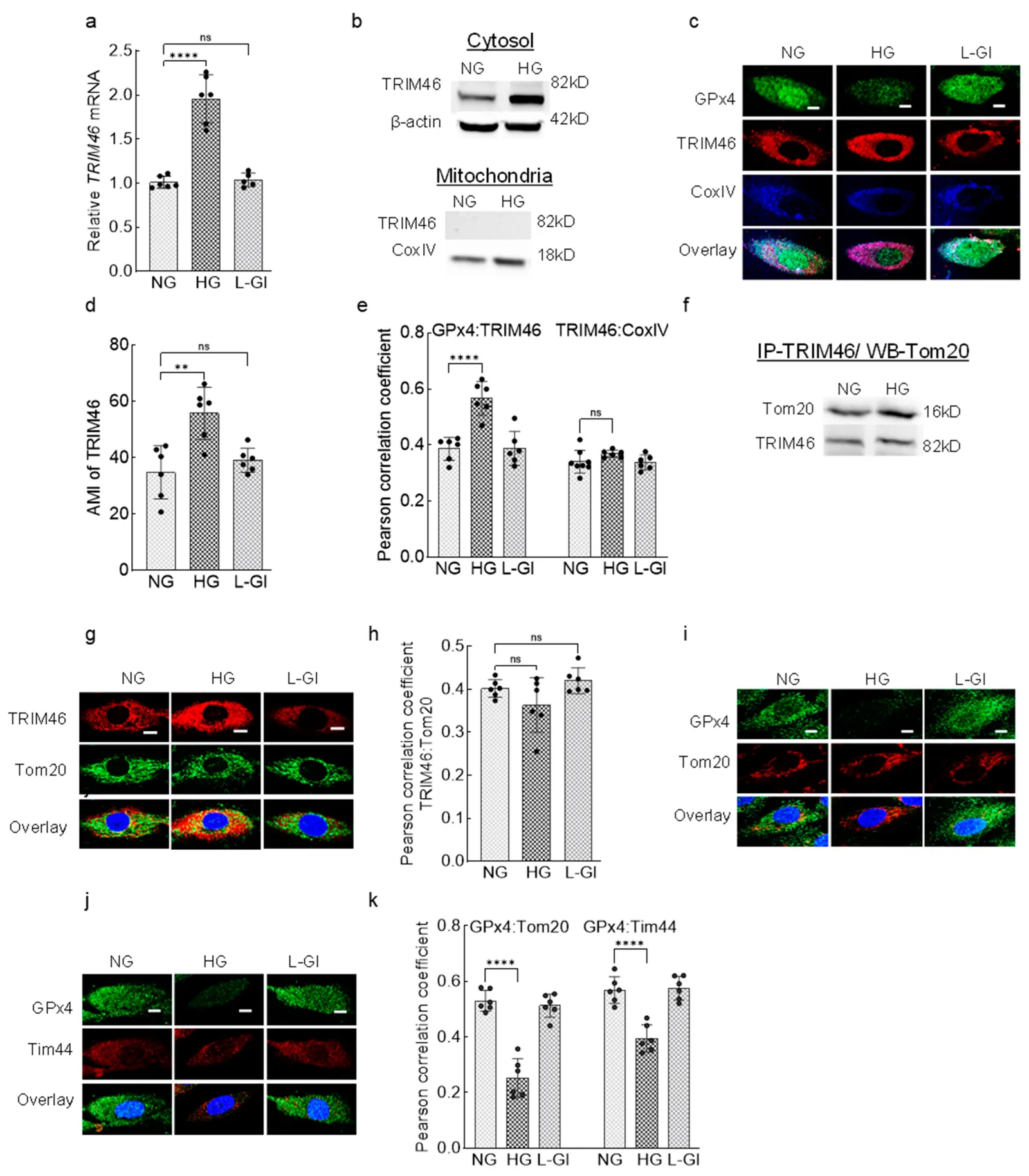
Mitochondrial localization of TRIM46 and GPx4. HRECs were analyzed for TRIM46 (**a**) mRNA using *β-actin* as a housekeeping gene, and (**b**) cytosolic and mitochondrial protein expression by Western blot technique using β-actin and CoxIV as their respective loading controls. (**c**) Representative image of cells immunostained with GPx4 (green), TRIM46 (red), and CoxIV (blue), and plots showing (**d**) AMI of TRIM46, and (**e**) Pearson correlation coefficient between GPx4-TRIM46 and TRIM46-CoxIV. (**f**) Binding of TRIM46 with Tom20 was determined by co-IP technique by western blotting Tom20 in TRIM46 immunoprecipitated HRECs. (**g**) Representative image of TRIM46-Tom20 interactions using the immunohistochemical technique: red indicates TRIM46, green Tom20, and blue DAPI, and (**h**) plot showing Pearson correlation coefficient between TRIM46-Tom20. Representative images of cells immunostained with (**i**) GPx4 (green) and Tom20 (red) and (**j**) GPx4 (green) and Tim44 (red), mounted using DAPI (blue) containing mounting medium. (**k**) Graph showing Pearson correlation coefficients between GPx4-Tom20 and GPx4-Tim44. Scale bar = 10 µm; NG = 5 mM D-glucose; HG = 20 mM D-glucose; L-Gl = 20 mM L-glucose. ** *p* < 0.01; **** *p* < 0.0001; ns = *p* > 0.05.

**Figure 3:**
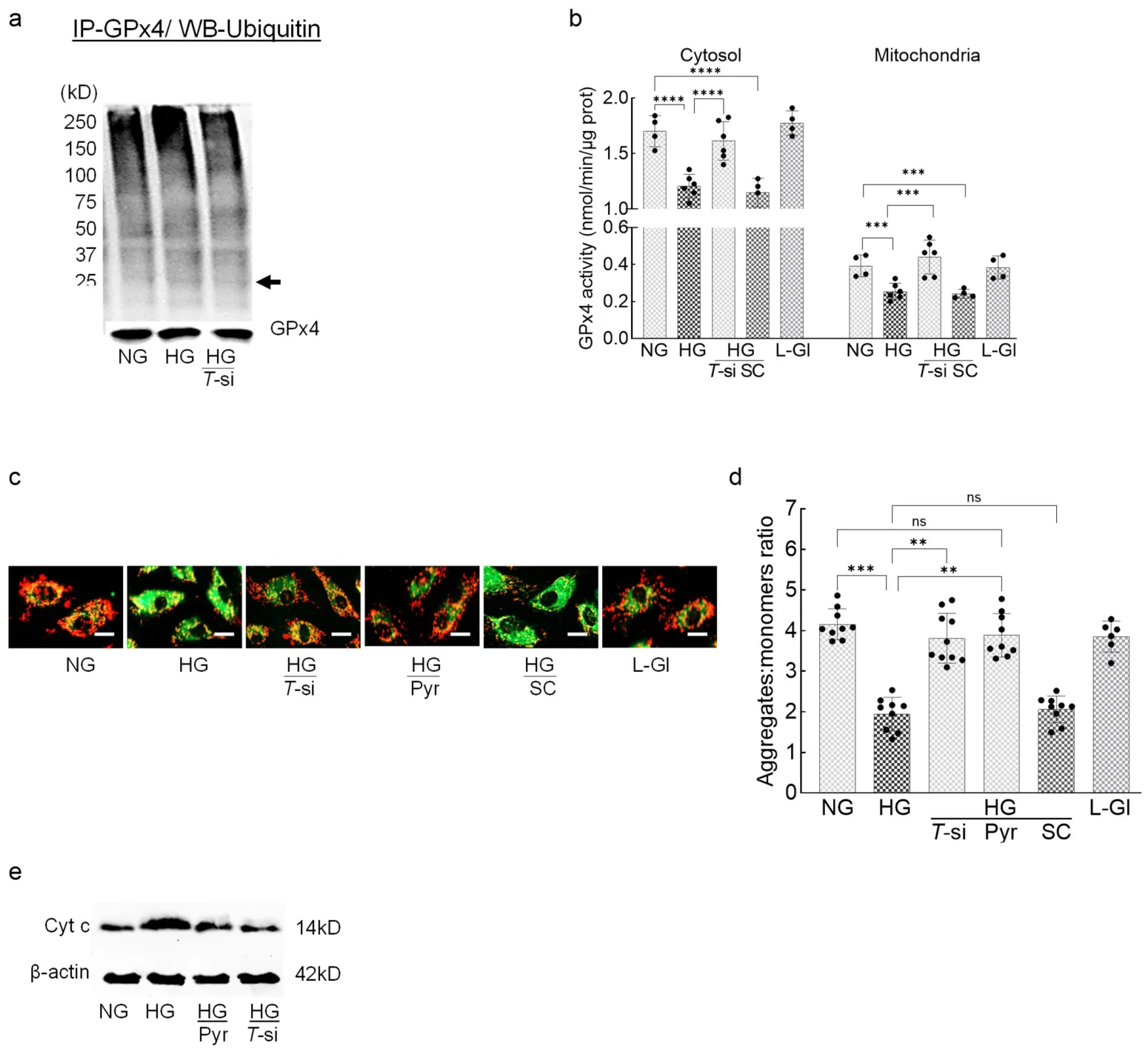
Regulation of TRIM46 and GPx4 ubiquitination. (**a**) GPx4 immunoprecipitated cell lysate was western blotted for ubiquitin using GPx4 as a loading control; the arrow indicates the GPx4 band. (**b**) Activity of GPx4 was quantified in the cytosolic and mitochondrial fractions. (**c**) Mitochondrial membrane potential was assessed by JC-1 staining, and (**d**) the graph shows the ratio of aggregates to monomers. (**e**) Western blot showing cytochrome c (Cytc) release in the cytosol; β-actin was used as the loading control. Scale bar = 20 µm; NG = 5 mM D-glucose; HG = 20 mM D-glucose; HG/*T*-si and HG/SC = *TRIM46*-siRNA or scrambled RNA-transfected cells in HG; HG/Pyr = HRECs pre-incubated with PYR 41, followed by incubation in 20 mM D-glucose; L-Gl = 20 mM L-glucose. ** *p* < 0.01; *** *p* < 0.001; **** *p* < 0.0001, ns = *p* > 0.05.

To understand the mechanism of GPx4 ubiquitination, the expression of TRIM46, an E-3 ligase, was analyzed. Consistent with GPx4 ubiquitination, TRIM46 gene transcripts and protein expression were increased by over 70% in cells in high glucose vs. normal glucose (Figure 2a,b). Immunocytochemical analysis also clearly showed a significant increase in TRIM46 fluorescence and AMI in high glucose vs. normal glucose (Figure 2c,d). Consistent with increased ubiquitination, the Pearson correlation coefficient between GPx4-TRIM46, although weak in cells in normal glucose or in L-glucose (<0.03), was strong in high glucose (~0.6), implying an increase in GPx4-TRIM46 interactions. However, the TRIM46-CoxIV correlation was weak in both normal and high glucose, suggesting poor TRIM46 mitochondrial import (Figure 2e). Since mitochondria are double-membrane structures and use membrane transporters to import proteins from the cytosol, and diabetes damages the retinal mitochondrial transport system [23], to further investigate whether TRIM46 is imported into mitochondria, its interactions with the outer membrane transporter were investigated. As shown by Co-IP, glucose had no effect on TRIM46 interaction with the outer membrane transporter Tom20, which was further confirmed by immunocytochemical technique with a weak correlation coefficient between TRIM46-Tom20 in cells in normal or high glucose (Figure 2f–h). Furthermore, despite the presence of heavy bands for mitochondrial marker CoxIV, mitochondrial protein western blots had an undetectable/very weak TRIM46 band in both normal- and high-glucose-incubated cells, further confirming lack/poor TRIM46 import inside the mitochondria (Figure 2b). To investigate whether glucose impaired mitochondrial import of ubiquitinated GPx4, its interaction with mitochondrial transporters was examined. High glucose significantly decreased GPx4 interactions with both Tom20 and Tim44, and its correlation coefficient with Tom20 and Tim44 was weak (<0.4) in high glucose vs. normal glucose or 20 mM L-glucose (Figure 2i–k). Co-IP data also indicated glucose-induced increased GPx4 ubiquitination, which was attenuated by *TRIM46*-siRNA. Consistently, the high glucose-induced decrease in GPx4 activity was also restored in both cytosolic and mitochondrial fractions by *TRIM46*-siRNA, but not in the scrambled control RNA group (Figure 3a,b). Mitochondrial function was also improved by both *TRIM46*-siRNA and a chemical inhibitor of ubiquitination, as indicated by restoration of mitochondrial membrane potential and inhibition of cytochrome c release in the cytosol (Figure 3c–e).

**Figure 4:**
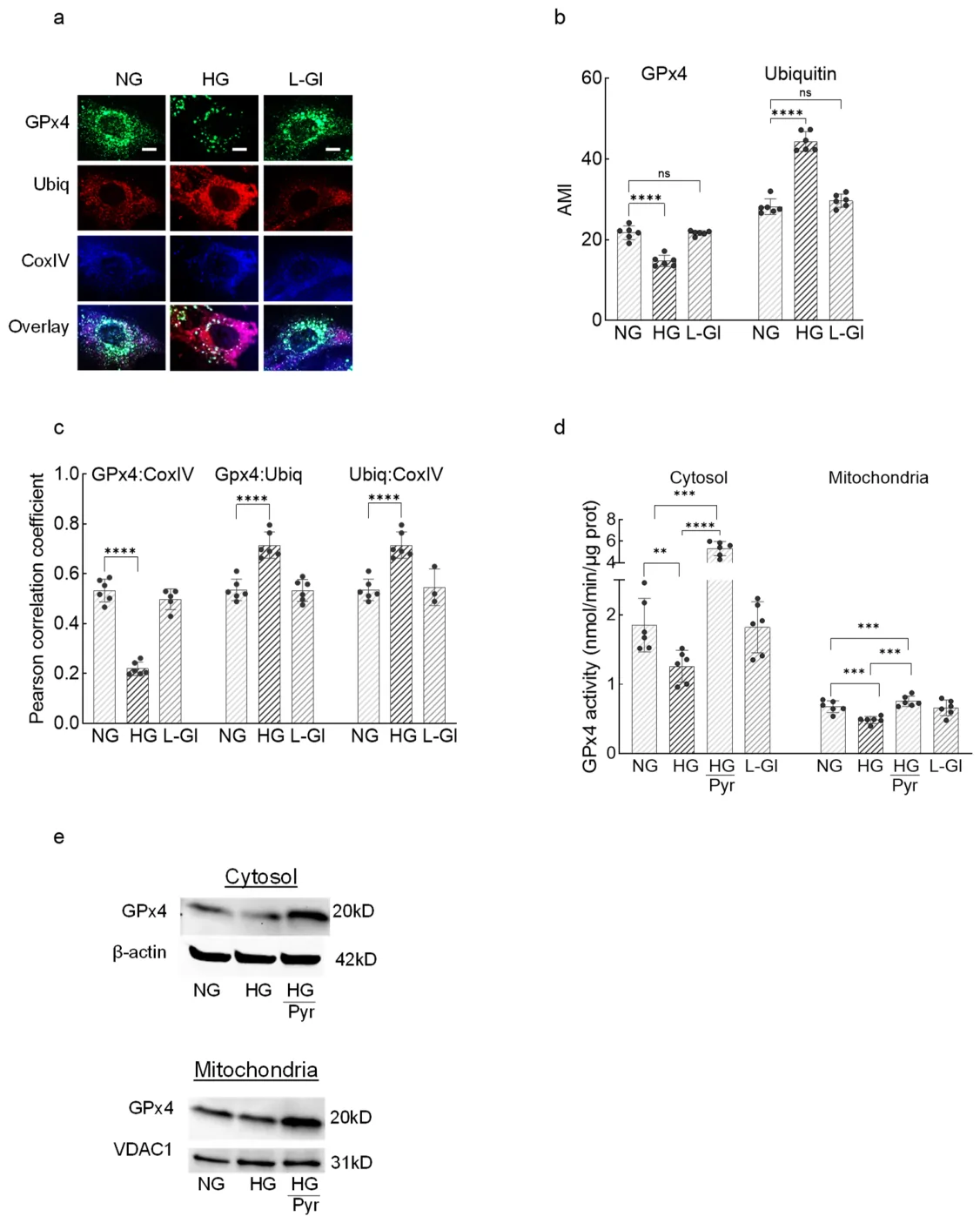
GPx4 and its ubiquitination in RMCs. RMCs incubated in high glucose for 96 h were analyzed for (**a**) mitochondrial localization and ubiquitination of GPx4. Representative immunofluorescence image of RMCs, captured with a 63× objective showing GPx4 (green), ubiquitin (red), and CoxIV (blue), and graphs displaying (**b**) AMI of GPx4 and ubiquitin, and (**c**) Pearson correlation coefficient between GPx4-CoxIV, GPx4-ubiquitin, and ubiquitin-CoxIV. (**d**) Activity of GPx4 was measured in cytosolic and mitochondrial fractions. (**e**) Protein expression of GPx4 was quantified in the cytosol and isolated mitochondria by western blot technique; β-actin and VDAC1 were used as their respective loading controls. Values are from 3–4 different experiments and are presented as mean ± SD. Scale bar = 10 µm; NG and HG = 5 mM or 20 mM D-glucose, respectively; L-Gl = 20 mM L-glucose; HG/Pyr = RMCs, pre-incubated with PYR 41, followed by incubation in 20 mM D-glucose. Values from 3-4 different experiments are presented as mean ± SD. ** *p* < 0.01; *** *p* < 0.001; **** *p* < 0.0001; ns = *p* > 0.05.

Human retinal Müller cells (RMCs): Consistent with retinal endothelial cells, GPx4 expression in the cytosol and mitochondria was significantly less in RMCs in high glucose vs. normal glucose; AMI of GPx4 was decreased by ~40%, and although Pearson correlation coefficient between GPx4-CoxIV was strong (>0.5) in normal glucose or L-glucose, it was <0.25 in high glucose (Figure 4a–c). Ubiquitin expression and the correlation coefficient of GPx4-ubiquitin were two-fold and ~30% higher, respectively, in RMCs in high glucose vs. normal glucose. Similarly, the correlation coefficient between ubiquitin-CoxIV was also significantly higher in cells in high glucose, suggesting increased ubiquitin in the mitochondria (Figure 4c), and PYR prevented glucose-induced GPx4 inhibition in both cytosol and mitochondria (Figure 4d). As shown by the Western blot image, PYR also restored cytosolic and mitochondrial GPx4 levels (Figure 4e).

**Figure 5:**
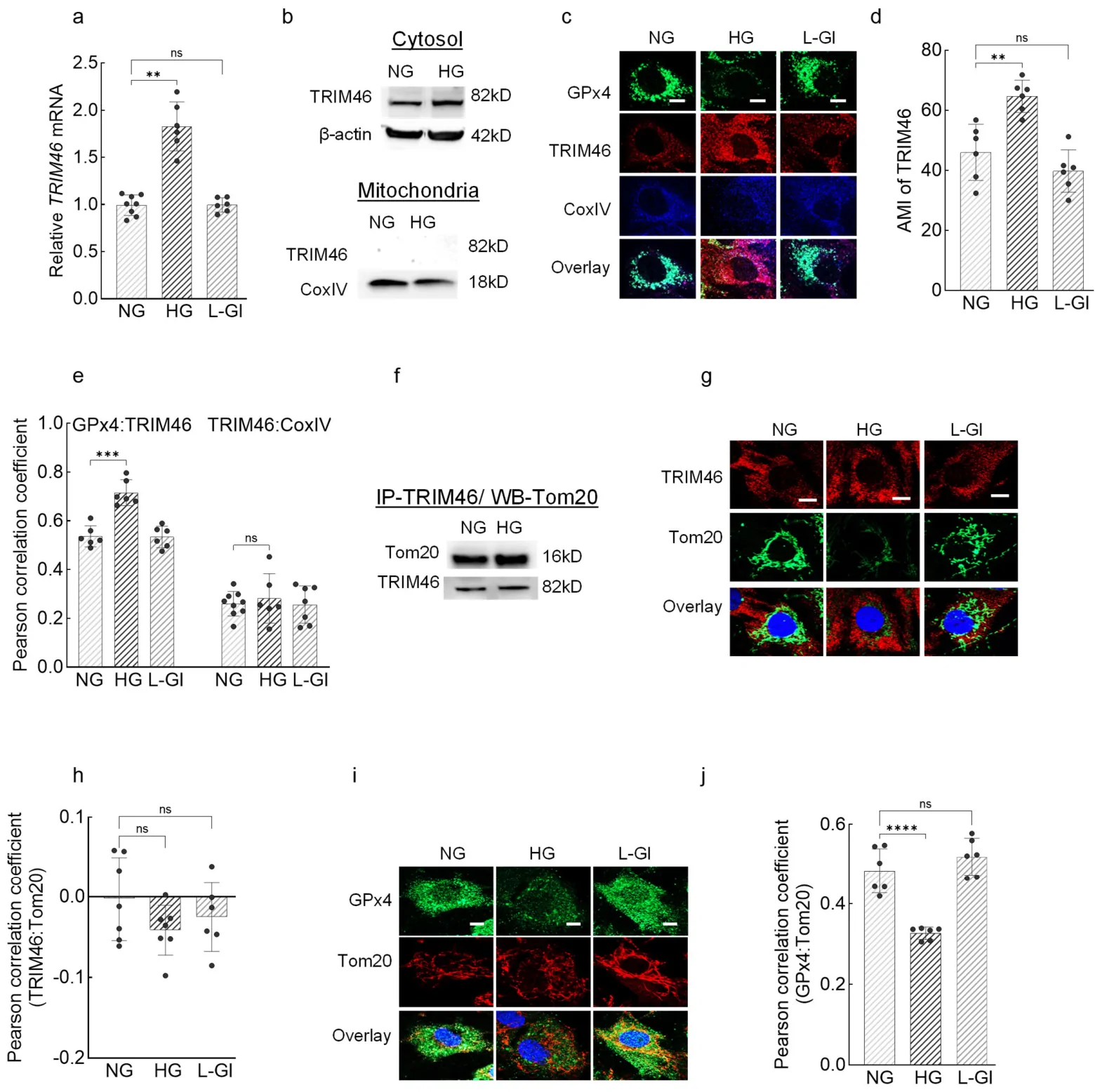
Effect of high glucose on mitochondrial localization of TRIM46 and GPx4. TRIM46 (**a**) mRNA was quantified by qRT-PCR using *β-actin* as a housekeeping gene, and (**b**) its protein expression in the cytosol and mitochondria was assessed by Western blot technique using β-actin and CoxIV as their respective loading controls. (**c**) Representative image displaying RMCs immunostained for GPx4 (green), TRIM46 (red), and CoxIV (blue), and accompanying plots show (**d**) AMI of TRIM46 and (**e**) Pearson correlation coefficient between TRIM46-GPx4 and TRIM46-CoxIV. TRIM46-Tom20 interactions were quantified (**f**) by western blotting Tom20 in TRIM46-immunoprecipitated RMCs using TRIM46 as a loading control, and (**g**) by immunostaining for TRIM46 (red) and Tom20 (green); blue represents DAPI, and (**h**) the graph shows the Pearson correlation coefficient between TRIM46-Tom20. (**i**) Representative image of RMCs immunostained for GPx4 (green) and Tom20 (red), and (**j**) graph showing Pearson correlation coefficients between GPx4 and Tom20. Scale bar = 10 µm. NG = 5 mM D-glucose; HG = 20 mM D-glucose; L-Gl = 20 mM L-glucose. Values are from three different experiments and are presented as mean ± SD. ** *p* < 0.01; *** *p* < 0.001; **** *p* < 0.0001; ns = *p* > 0.05.

**Figure 6:**
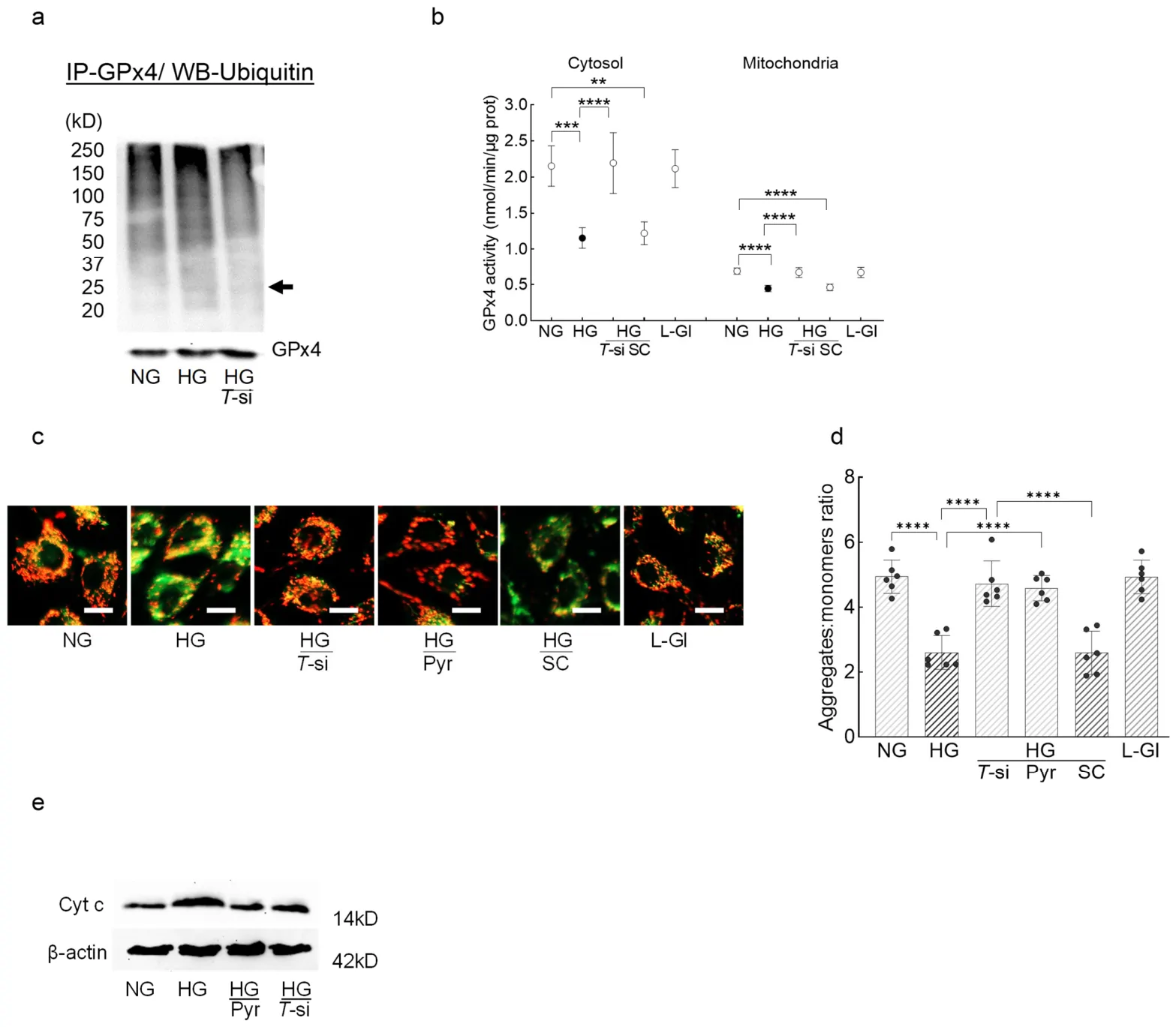
Effect of regulation of *TRIM46* on GPx4 ubiquitination in RMCs. (**a**) Cells Immunoprecipitated for GPx4 were western blotted for ubiquitin; the arrow indicates the GPx4 band. (**b**) Activity of GPx4 was measured in cytosolic and mitochondrial fractions. (**c**,**d**) RMCs stained with JC-1 displaying mitochondrial membrane potential, and the accompanying graph shows the ratio of aggregates to monomers. (**e**) Western blot showing cytochrome leakage in the cytosol. Scale bar = 20 µm. NG = 5 mM D-glucose; HG = 20 mM D-glucose; HG/*T*-si and HG/SC = *TRIM46*-siRNA or scrambled RNA transfected cells in HG; HG/Pyr = cells pre-incubated with PYR 41, followed by incubation in 20 mM D-glucose; L-Gl = 20 mM L-glucose. Values from three different experiments are presented as mean ± SD. ** *p* < 0.01; *** *p* < 0.001; **** *p* < 0.0001.

TRIM46 expression (gene and protein) increased by 1.5-2-fold; however, the correlation coefficient between TRIM46 and CoxIV remained very weak (<0.3), with no detectable TRIM46 in the mitochondria by western blotting, suggesting poor/no TRIM46 mitochondrial import (Figure 5a–e). TRIM46-Tom20 interactions, as determined by Co-IP (Figure 5f) and by immunocytochemical staining (Figure 5g,h), were similar in normal and high glucose, and the correlation between TRIM46 and Tom20 was negative. Furthermore, GPx4 interactions with Tom20 were also significantly reduced in RMCs in high glucose compared to normal glucose (Figure 5i,j). GPx4 ubiquitination was increased in high glucose, and *TRIM46*-siRNA prevented ubiquitination and inhibition of cytosolic and mitochondrial GPx4 activities (Figure 6a,b). Glucose-induced mitochondrial damage, including loss of mitochondrial membrane potential and cytochrome c release into the cytosol, was also attenuated by both *TRIM46*-siRNA and ubiquitination inhibitor PYR (Figure 6c–e). L-glucose had no effect on any of these parameters.

### 2.2. Mouse and Human Retina

**Figure 7:**
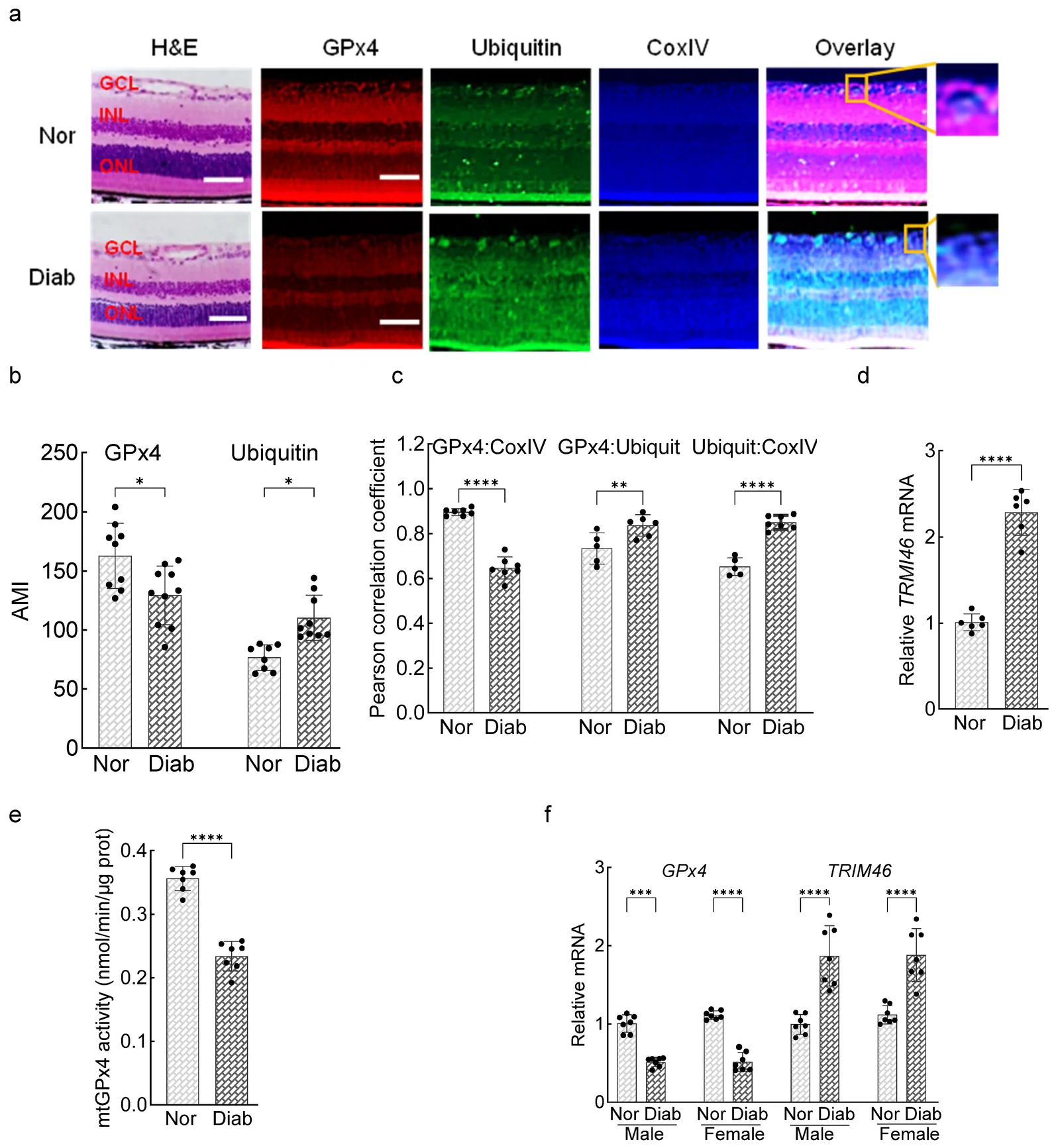
Ubiquitination of GPx4 in diabetic mouse retina. (**a**) Representative image of retinal section immunostained for GPx4 (red), ubiquitin (green), and CoxIV (blue); the left panel displays an H&E-stained section from the same retina; inserts are magnified. Graphs showing (**b**) AMI of GPx4 and ubiquitin, calculated in the entire retinal section, and (**c**) Pearson correlation coefficients between GPx4-CoxIV, GPx4-ubiquitin, and ubiquitin-CoxIV. (**d**) *TRIM46* mRNA in the retina was quantified by qRT-PCR, using *18S rRNA* as a housekeeping gene, and (**e**) GPx4 enzyme activity was measured in the isolated retinal mitochondria. (**f**) Graph showing relative retinal *GPx4* and *TRIM46* mRNA in male and female normal and diabetic mice. Values in the graphs are presented as mean ± SD from 5–6 mice/group. Nor and Diab = Normal and diabetic mice, respectively; Scale bar = 50 µm. GCL = ganglion cell layer; INL = inner nuclear layer; and ONL = outer nuclear layer. * *p* < 0.03; ** *p* < 0.01; *** *p* < 0.001; **** *p* < 0.0001.

To confirm the in vitro results, a retina from mice diabetic for 24 weeks was analyzed. Average blood glucose and average body weight for diabetic mice were 482 ± 73 mg/dL and 21 ± 1.4 g, respectively, compared to 130 ± 21 and 28 ± 2 for normal control mice. GPx4 staining was significantly reduced in the entire retinal layers in diabetic mice, and this was further supported by >45% decrease in GPx4 AMI. Furthermore, the Pearson correlation coefficient between GPx4 and CoxIV decreased, suggesting less mtGPx4 expression. This was accompanied by increased staining for ubiquitin and a correlation coefficient between GPx4-ubiquitin and ubiquitin-CoxIV (Figure 7a–c). *TRIM46* mRNA levels were significantly increased, and mtGPx4 activity was inhibited in the retina from diabetic mice vs. nondiabetic mice (Figure 7d,e). Figure 7f is included to show that the mouse gender did not influence the diabetes-induced decrease in GPx4 and increase in TRIM46 mRNA levels.

**Figure 8:**
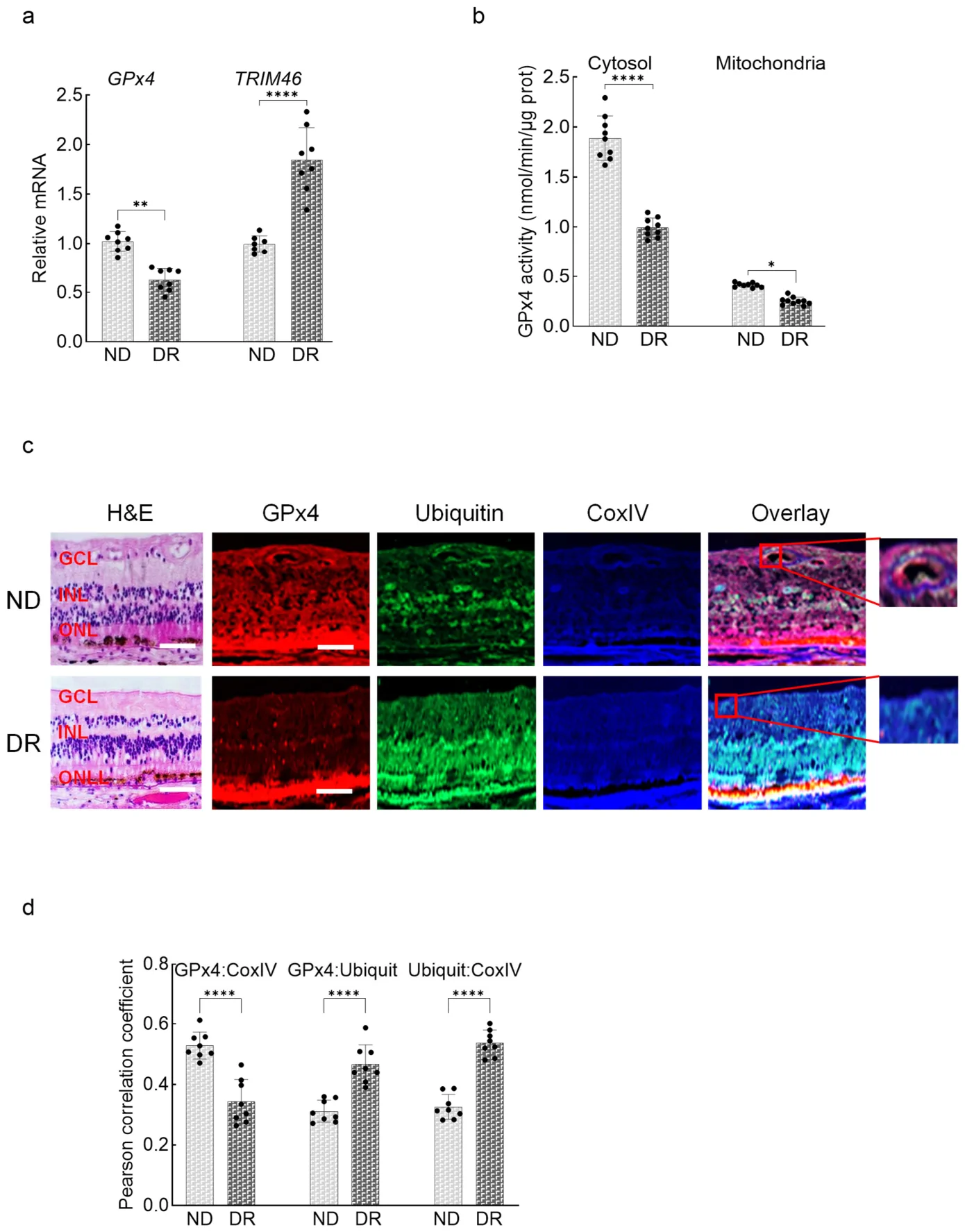
GPx4 and TRIM46 in retina from human donors: Human retinal (**a**) mRNA levels of *GPx4* and *TRIM46* were quantified by qRT-PCR, and (**b**) GPx4 enzyme activity was measured in cytosol and isolated mitochondria. (**c**) Representative retinal section, immunostained for GPx4 (red), ubiquitin (green), and CoxIV (blue); the left panel displays an H&E-stained section from the same retina; inserts show magnified areas. (**d**) Graph showing Pearson correlation between GPx4-CoxIV, GPx4-ubiquitin, and Ubiquitin-CoxIV. Values in the graphs are from 8–9 human donors/group and are presented as mean ± SD. ND and DR = Nondiabetic donor and diabetic donors with retinopathy, respectively; Scale bar = 50 µm. GCL = ganglion cell layer; INL = inner nuclear layer and ONL = outer nuclear layer. * *p* < 0.03; ** *p* < 0.01; **** *p* < 0.0001.

In accordance with results from the experimental models, compared to nondiabetic donors, gene transcripts and fluorescence intensity of GPx4 were significantly decreased, and *TRIM46* transcripts were increased in the retina from donors with diabetic retinopathy; cytosolic and mtGPx4 activities were inhibited by >30% (Figure 8a–c). Furthermore, the Pearson correlation coefficient between GPx4 and CoxIV was low in the DR group (<0.4), but between GPx4-ubiquitin and ubiquitin-CoxIV, it was significantly high (*p* < 0.0001, Figure 8d).

## 3. Discussion

Glutathione peroxidase 4 reduces phospholipid hydroperoxides to the corresponding lipid alcohols using GSH as a cofactor to prevent lipid peroxidation and limit membrane damage [12,13,24]. This enzyme has three isoforms-cytosolic, mitochondrial, and nuclear isoforms- and mtGPx4 has an N-terminus with a 27-amino-acid mitochondrial-targeting sequence, which is cleaved during mitochondrial import [25]. Recently, we have shown that in diabetes, while lipid peroxide levels are increased in the retina, GPx4 activity is decreased both in the cytosol and in mitochondria, leading to mitochondrial damage-cell ferroptosis [14]. Results presented here imply that hyperglycemia increases GPx4 ubiquitination, resulting in poor mitochondrial import of ubiquitinated GPx4, and this phenomenon is observed in both vascular (endothelial) and nonvascular (Müller) cells of the retina. Furthermore, E3 ubiquitinase TRIM46 plays a major role in GPx4 ubiquitination, and in the mitochondria, despite increased levels of ubiquitinated mtGPx4, TRIM46 levels are not increased. While the interaction of GPx4 with mitochondrial transporters, Tom20 and Tim44, is significantly decreased in hyperglycemia, TRIM46 interaction with Tom20 is not affected. These results imply that GPx4 is mainly ubiquitinated in the cytosol in a hyperglycemic milieu, and due to poor interactions of the ubiquitinated GPx4 with mitochondrial transporters, its import into the mitochondria is decreased.

Glutathione peroxidase 4 is considered a ‘scavenger’ of lipid peroxides, and this oxidoreductase acts as a sensor of oxidative stress and cell death signals [13,24,26]; mt-GPx4 prevents the release of cytochrome c and regulates apoptosis [12]. In the retina, mt-GPx4 is associated with photoreceptor development and survival [27]. In diabetes, retinal mt-GPx4 is inhibited, leading to accumulation of lipid peroxides and cell loss by ferroptosis [14]. Enzyme activity can be influenced by posttranslational modifications, the covalent modifications that change protein structure/activity following protein biosynthesis [28,29], and diabetes facilitates many post-transcriptional modifications, including phosphorylation, glycation, and ubiquitination [20,29,30,31]. The covalent binding of ubiquitin to the lysine residues of the protein triggers ubiquitination, a three-step enzymatic cascade, which involves ubiquitinase enzymes E1, E2, and E3, resulting in the transfer of ubiquitin, via its C-terminal glycine, onto the ε-amino group of a lysine residue on the substrate protein [32,33]. Ubiquitinated protein is eventually degraded by proteasomes to balance protein quantity and quality [16,29]. GPx4 can be ubiquitinated on Lys47, Lys80, Lys107, and Lys135 [26]. Here, we show that ubiquitination of GPx4 is increased in diabetes, and TRIM46, which promotes ubiquitination and proteasomal degradation of proteins, plays an important role. Furthermore, *TRIM46*-siRNA ameliorates glucose-induced GPx4 ubiquitination and its activity, suggesting a direct role of TRIM46 in GPx4 regulation in diabetes. In accordance with this, our results show that both *TRIM46*-siRNA and the ubiquitination inhibitor Pyr41 also prevent glucose-induced mitochondrial dysfunction. In accordance with our results, high glucose-induced TRIM46-mediated ubiquitination and degradation of NF-kB inhibitor IkBα is implicated in cytokine storms and hyperpermeability in retinal endothelial cells, and *TRIM46* silencing in endothelial cells is shown to reverse ferroptosis agonist-mediated cell loss [34]. In addition, increased TRIM46 levels in HK2 human renal proximal tubular epithelial cells under hypoxic conditions are shown to ubiquitinate and proteasomally degrade Axin1, a negative regulator of Wnt/β-catenin signaling activity [35,36].

Glutathione peroxidase 4 has a mitochondrial targeting sequence, and mt-GPx4 prevents release of cytochrome c and regulates apoptosis [12]. In the retina, mt-GPx4 is associated with photoreceptor development and survival [27]. In diabetes, retinal mt-GPx4 is inhibited, leading to accumulation of lipid peroxides and cell loss by ferroptosis [14]. TRIM46 is not a resident mitochondrial protein, which is supported by western blotting of the mitochondrial fraction showing a low to negligible TRIM46 band. However, immunohistochemical analysis has displayed low (<0.04) Pearson correlation coefficients between TRIM46:CoxIV, TRIM46:Tom20, and TRIM46:Tim44, and co-IP analysis has exhibited some association of TRIM46 with Tom20. The exact reason for such association is not clear, but high glucose conditions could be facilitating recruitment of TRIM46 on the mitochondrial surface, where it might be interacting with the mitochondrial import machinery and ubiquitinating GPx4.

Thus, overall, our results suggest that in hyperglycemia, mitochondria themselves might not be ubiquitinating GPx4; instead, GPx4 ubiquitinated in the cytosol could be imported into the mitochondria. In addition, our results indicate that a ubiquitination inhibitor ameliorates the high glucose-induced decrease in GPx4 levels in both the cytosol and mitochondria, further supporting TRIM46-mediated GPx4 ubiquitination. The role of other posttranslational mechanisms, including succination and methylation, regulating GPx4 activity, and conformational changes mediated by binding of compounds to the GPx4 allosteric site, affecting its enzymatic functions, however, cannot be ruled out [37,38]. Furthermore, GSH is an obligatory cofactor for GPx4 activation, which is regulated by the master regulator Nrf2 and cystine/glutamate antiporter, System X_c_^−^ [39,40]; role of these important alternative mechanisms in regulating GPx4 activity in diabetic retinopathy, however, requires further experimental validation.

Conventionally, diabetic retinopathy is predominantly considered as a microvascular disease, but retinal nonvascular cells, including ganglion cells and Müller glial cells, are also lost in diabetes [5,41,42,43]. Müller cells, the principal glial cells that expand radially across almost the whole width of the retina, enveloping neural synapses and surrounding retinal capillaries [21,22], interact with nearly the entire retinal cell population, including endothelial cells, and provide metabolic support, helping maintain the blood-retinal barrier and antioxidant activity [44,45]. In diabetic retinopathy, both endothelial cells and Müller cells show changes in their morphology and co-regulate the inner blood–retinal barrier, and a complex interplay between Müller cells and endothelial cells is also implicated in neovascularization in diabetic retinopathy [46]. Results from retinal Müller cells show that, in accordance with endothelial cells, high glucose also ubiquitinates GPx4 and inhibits its cytosolic and mitochondrial activity, and ubiquitinated GPx4 is poorly imported into the mitochondria.

Mouse model and retina from human donors also present similar results as obtained from the in vitro models; compared to nondiabetic controls, retinal cytosol and mitochondria show decreased GPx4 activity in diabetic mice and in human donors with documented diabetic retinopathy, and TRIM46 and ubiquitination of GPx4 are increased in the entire retinal layers. The correlation coefficient between GPx4 and CoxIV is decreased, but the correlation coefficient between ubiquitin and CoxIV is increased, further supporting the importance of TRIM46-mediated GPx4 ubiquitination in diabetes. Diabetes affects the entire retinal cell population; these results validate that the TRIM46-GPx4 pathway is also affected in most of the retinal cellular components. However, we recognize that co-immunostaining with endothelial and Müller cell markers would be desirable to further confirm our in vitro results, but limited sample availability for this study has made it challenging to perform co-immunostaining.

Consistent results from in vitro and in vivo models of diabetic retinopathy are presented here, but we recognize that in vitro and in vivo models may not fully reproduce the complexity and heterogeneity of human diabetic retinopathy. To support translational relevance, we have also analyzed retina from human donors with documented diabetic retinopathy; however, our small sample size (8–9 donors/group) does not allow us to exclude the potential selection bias related to donor variability. Moreover, there remains a possibility that biochemical assays (GPx4 activity, ubiquitination, and protein-protein interactions) could be affected by technical variability, including efficiency of mitochondrial fractionation and antibody specificity. Our data support TRIM46-mediated ubiquitination/inhibition of GPx4; however, we cannot rule out the role of other E3 ligases in GPx4 ubiquitination.

In conclusion, our data from retinal vascular and nonvascular cells, a diabetic mouse model, and human samples with diabetic retinopathy imply that, due to increased TRIM46 in diabetes, GPx4 in the cytosol is ubiquitinated, and interactions of ubiquitinated GPx4 with mitochondrial membrane transporters are impaired, resulting in poor import of GPx4 into mitochondria. This results in the inhibition of mtGPx4 and mitochondrial dysfunction, ultimately leading to cell death and development of diabetic retinopathy. However, whether ubiquitination directly impairs cytosolic GPx4 import into mitochondria needs further investigation.

## 4. Materials and Methods

### 4.1. Retinal Endothelial Cells

HRECs (primary cells; Cat no. ACBRI 181; Cell Systems Corp., Kirkland, WA, USA) from passages 6th–8th were incubated for 96 h in Dulbecco’s Modified Eagle Medium (DMEM) supplemented with 1% heat-inactivated fetal bovine serum (FBS), 9% Nu-serum, and 1 µg/mL endothelial growth supplement containing either 5 mM D-glucose (normal glucose) or 20 mM D-glucose (high glucose). To investigate the role of ubiquitination, cells were pre-treated with 5 µM Ubiquitin E1 inhibitor, PYR 41 (4-[4-[(5-nitro-2-furanyl)methylene]-3,5-dioxo-1-pyrazolidinyl]-benzoic acid, ethyl ester), Cat# N291, Sigma-Aldrich, St. Louis, MO, USA) for 4 h; PYR 41 concentration is based on reports from others and our laboratory demonstrating effective inhibition of ubiquitination, with minimal cytotoxicity [20,47]. Each experiment included an osmotic/metabolic control where cells were incubated in 20 mM L-glucose (L-Gl) [7,48].

### 4.2. Retinal Müller Cells

Primary human RMCs (Cat no. ABT-TC133L, Accegen, Fairfield, NJ, USA) from 6th–8th passage were incubated for 96 h in DMEM containing 2% FBS, 8% Nu-Serum, and 1% antibiotic/antimycotic solution, supplemented with either 5 mM or 20 mM D-glucose [48], in the absence or presence of PYR 41. As with HRECs, RMCs in 20 mM L-glucose served as osmotic/metabolic control.

A group of HRECs or RMCs from 5th–6th passage were transfected with *TRIM46*-siRNA (*T*-si; Cat. No. 4392420, ID s36934, Invitrogen, Carlsbad, CA, USA) or scrambled RNA control (SC) using Lipofectamine RNAiMAX transfection reagent (Cat. No. 13778-030; Invitrogen, USA). The efficiency of knockdown, assessed by quantifying *TRIM46* mRNA, was 55–60%.

### 4.3. Mice

C57BL/6J mice (20–25 g BW, male and female; Jackson Laboratory, Bar Harbor, ME, USA) were made diabetic by injecting 55 mg/kg streptozotocin (i.p.) for four consecutive days [7,14]. Three days after the last streptozotocin injection, mice with blood glucose >250 mg/dL were considered diabetic. Age- and sex-matched non-diabetic mice served as their controls. Mice had free access to water and food and were housed in a temperature- and humidity-controlled room with a 12 h light/dark cycle. They were monitored daily, and their body weight and blood glucose were measured every two weeks. Both male and female streptozotocin-induced diabetic mice had similar blood glucose values. Animals were sacrificed 24 weeks after induction of diabetes by CO_2_ overdose and cervical dislocation, and their eye globes were either fixed in alcoholic Z-Fix (Excalibur Pathology, Norman, OK, USA) for paraffin sectioning and subsequent hematoxylin and eosin staining [49] or were used to harvest retina for biochemical/molecular measurements. Twenty-four weeks of Stz-induced diabetes was chosen because, at this duration, retinal histopathology characteristic of diabetic retinopathy, including degenerative capillaries and pericyte ghosts, can be seen. These animal protocols, designed to minimize pain or discomfort to the animals, were approved by the Animal Care and Use Committee of Wayne State University and followed the guidelines of the Association for Research in Vision and Ophthalmology Resolution on the Use of Animals in Research.

### 4.4. Human

Human post-mortem eye globes from nine donors with diabetes for >20 years and clinically documented retinopathy, nucleated within 6–9 h after death, were obtained from the Eversight Eye Bank (Ann Arbor, MI, USA). Either the whole eye globe was fixed for immunohistochemistry in the modified Davidson’s fixative containing 30% of a 37% formaldehyde, 15% ethanol, 5% glacial acetic acid, and 50% distilled water [50], or the retina was harvested. Nondiabetic donors with no history of ocular or systemic diseases served as controls [51]. While the diabetic retinopathy group had 50% males with an average age of 64 ± 7 years, the control group had 60% males with an average age of 62 ± 13 years. Due to limitations in obtaining donors’ medical records, detailed ophthalmologic grading, e.g., NPDR or PDR stage, was not available. All eye globes, collected postmortem by the Eye Bank, were supplied without patient-identifying information, and the use of the tissues was exempt from Institutional Review Board Approval.

### 4.5. Isolation of Mitochondria and Cytosol

Cells/retina were homogenized in mitochondrial isolation buffer containing 25 mM Tris-HCl (pH 7.4), 250 mM sucrose, 2 mM EDTA, and 1 µg/mL of a protease inhibitor cocktail, and after removing cell debris, it was centrifuged at 10,000× *g* for 15 min. The resulting mitochondrial pellet was washed with the same buffer [7] and resuspended in the RIPA lysis buffer for Western blotting or GPx4 assay buffer (100 mM potassium phosphate (pH 7.4), 150 mM KCl, 0.05% CHAPS, 5 mM β-mercaptoethanol, and 1 µg/mL protease inhibitor cocktail) for activity. The cytosolic fraction was prepared by centrifuging the supernatant at 100,000× *g* for 60 min and was used to measure cytochrome c release by Western blotting [14].

### 4.6. GPx4 Activity

In 100 µL assay buffer containing 5 mM EDTA, 5 mM reduced glutathione, 0.1% Triton X-100, 180 IU/mL glutathione reductase, and 160 mM NADPH/H^+^ (pH 7.8), and 20 µg protein (cytosol/mitochondria), after adding 30 mM (5 µL) cumene hydroperoxide, absorbance at 340 nm was recorded for five minutes. GPx4 activity was expressed as nmols/minute/µg protein [14,52].

### 4.7. Gene Expression

Trizol-extracted RNA was reverse transcribed into cDNA using High-Capacity cDNA Reverse Transcription Kit (Cat no. 4368814, Applied Biosystems, Waltham, MA, USA). Gene transcripts were quantified in triplicates by real time quantitative PCR (qRT-PCR) using SYBR green master mix and gene- and species-specific primers (Sequences 5′-3′; Human: *TRIM46* Fwd-5′CTGCTTGAGAACCCCGAC, Rev-GCTCGCTGGTGCTTGCTG; *GPx4* Fwd-TGGACGAGGGGAGGAGCC, Rev-CGATGTCCTTGGCGGAAAAC; *β-actin* Fwd-AGCCTCGCCTTTGCCGATCCG, Rev-TCTCTTGCTCTGGGCCTCGTCG; Mouse: *TRIM46* Fwd-AGTCCGCATCAGTACAAGCA, Rev-GCACACGTTGTGGGTACAAG; *GPx4* Fwd-CCGTCTGAGCCGCTTACTTAA, Rev-TGACGATGCACACGAAACC; *18S rRNA* Fwd-GCCCTGTAATTGGAATGAGTCCACTT, Rev-CTCCCCAAGATCCAACTACGAGCTTT). Relative gene expression was calculated by the delta-delta Ct method using either *β-actin* (human) or *18S rRNA* (mouse) as housekeeping genes [14,48].

### 4.8. Western Blotting

Protein (50 µg) was separated on a 4–15% SDS-polyacrylamide electrophoresis gel and transferred to nitrocellulose membranes. After blocking with 5% nonfat milk, the membranes were incubated with primary antibodies for TRIM46 (Cat no. 307967, Abcam, Cambridge, MA, USA; 1:150 dilution), GPx4 (Cat no. 67763-1-Ig, Proteintech, Rosemont, IL, USA, 1:500 Dilution), or cytochrome c (Cat no. AB13575, Abcam, 1:500 Dilution). Membranes were washed with Tris-buffered saline containing 0.1% Tween-20 and incubated with sheep anti-mouse IgG-peroxidase (Cat no. A5906, Sigma-Aldrich; 1:500 dilution) or sheep anti-rabbit IgG-peroxidase (Cat no. A6154, Sigma-Aldrich; 1:500 dilution), and the bands were detected by the chemiluminescent HRP substrate (Cat no.WBKLS0050, Millipore, Burlington, MA, USA). β-actin (Cat no. A5441, Sigma-Aldrich; 1:500 dilution) was used as loading protein and VDAC1 (Cat no. 66345-1-Ig, Proteintech; 1:500 dilution) or CoxIV (Cat no. 66110-1-Ig, Proteintech; 1:500 dilution) as mitochondrial markers [14].

### 4.9. Co-Immunoprecipitation

Cells were homogenized in lysis buffer containing 10 mM EGTA, 5 mM EDTA, 1% Triton X-100, 250 mM sucrose, 1 mM NaF, 1 mM phenylmethylsulfonyl fluoride, 1 mM Na_3_VO_4_, and protease inhibitors. Protein (100 µg) was incubated overnight at 4 °C with either 1 µg of anti-GPx4 (Cat no. 67763-1-Ig, Proteintech) or anti-TRIM46 antibody (Cat no. 307967, Abcam), and the immune complexes were captured by incubation with 20 µL pre-washed protein A/G agarose beads (Cat no. sc-2003, Santa Cruz Biotechnology, Dallas, TX, USA). Beads were washed with lysis buffer, and the bound proteins were eluted by boiling in SDS sample loading buffer. Proteins were separated on SDS-PAGE, and the membranes were immunoblotted with 1:500 diluted antibodies against Ubiquitin (Cat no.PA1-10023, Invitrogen), GPx4 (Cat no. 67763-1-Ig, Proteintech), Tom20 (Cat no. 118021-1-AP, Proteintech), and TRIM46 (Cat no. 307967, Abcam) [20].

### 4.10. Immunocytochemistry

Cells fixed in 4% paraformaldehyde were washed with PBS and permeabilized with 0.5% Triton X-100. After blocking with 5% BSA, they were incubated with antibody against GPx4 (Cat no. 67763-1-Ig, Proteintech), Ubiquitin (Cat no. PA1-10023, Invitrogen), CoxIV (Cat no. AB63947, Abcam), TRIM46 (Cat no. 307967, Abcam), mitochondrial outer membrane transporter Tom20 (Cat no. 118021-1-AP, Proteintech) or mitochondrial inner membrane transporter Tim44 (Cat no. PA5-106383, Invitrogen), each at 1:1000 dilution. After washing with PBS, cells were incubated with either TexasRed-conjugated anti-rabbit (Cat no. TI-1000, Vector laboratories, Burlingame, CA, USA), DyLight 488-conjugated anti-mouse (Cat no. DI-2488, Vector laboratories) or DyLight 405-conjugated anti-goat (Cat no.AB175664, Abcam) secondary antibodies, each at 1:1000 dilution, and the coverslips were mounted using Vectashield mounting medium with/without DAPI (Cat no.H2000 and H-1000, respectively, Vector laboratories). Images were captured using a Zeiss Apotome fluorescence microscope(Zeiss, Oberkochen, Germany) at 63× magnification [14,48]. Each experiment included an IgG isotype control where, instead of primary antibodies, IgG control antibodies were used. Arithmetic mean intensity of each fluorescence channel (red, green, and blue) was quantified on a per-cell basis using ZEN 2.6 pro software (version 2.6.76.0000) associated with the Zeiss Apotome 2 microscope. For colocalization analysis, Pearson correlation coefficients were calculated between the indicated pairs of fluorescence channels using ZEN 2.6 pro software (version 2.6.76.0000).

### 4.11. Mitochondrial Membrane Potential

Cells were incubated with 5 μM JC-1 (Cat. No. MP03168, Molecular Probes, Carlsbad, CA, USA) for 30 min at 37 °C, washed with PBS, and then imaged using a Zeiss microscope (20× objective). Fluorescence of red J-aggregates and green monomers was quantified, and the aggregate-to-monomer ratio was calculated to evaluate mitochondrial membrane potential [14].

### 4.12. Immunohistochemistry

Retinal paraffin sections (10 µm thick) were permeabilized with 0.5% triton X-100 for 10 min and blocked in 3% BSA containing 5% goat serum before incubating with antibody against GPx4 (Cat no. 67763-1-Ig, Proteintech), Ubiquitin (Cat no. PA1-10023, Invitrogen) and CoxIV (Cat no. AB63947, Abcam); each at 1:100 dilution. After washing with PBS, sections were incubated with corresponding-TexasRed-conjugated anti-mouse (Cat no.TI-1000, Vector Laboratories), TexasRed-conjugated anti-rabbit (Cat no.TI-1000, Vector Laboratories), DyLight 488-conjugated anti-rabbit (Cat no. DI-1488, Vector Laboratories), or DyLight 405-conjugated anti-goat (Cat no. AB175664, Abcam) secondary antibodies, diluted 1:250. The slides were mounted in Vectashield mounting medium, and the images were acquired by a Zeiss ApoTome fluorescence microscope using a 20× objective. For structural analysis, hematoxylin and eosin (H&E) stained retinal sections were also imaged [53].

### 4.13. Statistical Analysis

Data represent multiple biological replicates, with each measurement performed in triplicate. Statistical analysis was performed using GraphPad Prism (version 10.3.1, San Diego, CA, USA). Data distribution normality was assessed using the Shapiro-Wilk test. For normally distributed data, one-way ANOVA followed by Tukey’s post-hoc test was used, while non-normally distributed data were analyzed using the Kruskal–Wallis test followed by Dunn’s post hoc multiple comparisons test, and *p* values < 0.05 were considered significant.

## Data Availability

R.A.K. is the guarantor of this work and, as such, has full access to all the data in the study and takes responsibility for the integrity of the data and the accuracy of the data analysis. The data presented in this study are available on request from the corresponding author.
